# ZnO Nanocomposites of *Juniperus procera* and *Dodonaea viscosa* Extracts as Antiproliferative and Antimicrobial Agents

**DOI:** 10.3390/nano12040664

**Published:** 2022-02-16

**Authors:** Maha D. Alghamdi, Syed Nazreen, Nada M. Ali, Touseef Amna

**Affiliations:** 1 Chemistry Department, Faculty of Science, Albaha University, P.O. Box 1988, Albaha 65799, Saudi Arabia; mahaalghamdi@bu.edu.sa (M.D.A.); nada.m@bu.edu.sa (N.M.A.); 2 Department of Biology, Faculty of Science, Albaha University, P.O. Box 1988, Albaha 65799, Saudi Arabia

**Keywords:** nanocomposite, *Juniperus procera*, *Dodonaea viscosa*, anticancer, antibacterial

## Abstract

Cancer and microbial infections constitute a major burden and leading cause of death globally. The development of therapeutic compounds from natural products is considered a cornerstone in drug discovery. Therefore, in the present study, the ethanolic extract and the fractions of *Dodonaea viscosa* and *Juniperus procera* were evaluated for anticancer and antimicrobial activities. It was found that two fractions, JM and DC, exhibited promising anticancer and antimicrobial activities. The JM and DC fractions were further modified into ZnO nanocomposites, which were characterized by SEM, XRD, TGA, and EDX. It was noted that the synthesized nanocomposites displayed remarkable enhancement in cytotoxicity as well as antibacterial activity. Nanocomposite DC–ZnO NRs exhibited cytotoxicity with IC_50_ values of 16.4 ± 4 (HepG2) and 29.07 ± 2.7 μg/mL (HCT-116) and JM–ZnO NRs with IC_50_ values of 12.2 ± 10.27 (HepG2) and 24.1 ± 3.0 μg/mL (HCT-116). In addition, nanocomposites of DC (i.e., DC–ZnO NRs) and JM (i.e., JM–ZnO NRs) displayed excellent antimicrobial activity against *Staphylococcus aureus* with MICs of 2.5 and 1.25 μg/mL, respectively. Moreover, these fractions and nanocomposites were tested for cytotoxicity against normal fibroblasts and were found to be non-toxic. GC-MS analysis of the active fractions were also carried out to discover the possible phytochemicals that are responsible for these activities.

## 1. Introduction

Despite technological and medical developments, cancer and microbial infections constitute a major burden and leading cause of death globally [1]. Due to the impaired immunity in cancer patients, microbial infection is more prevalent than in non-cancer patients [2]. Moreover, the treatment of these diseases has many downfalls in terms of toxicity, selectivity, resistance, and non-differentiation between normal and cancerous cells [3]. Therefore, it is highly desirable to develop new anticancer and antimicrobial agents with minimal side effects and excellent selectivity.

The development of therapeutic compounds from natural products is considered a cornerstone in drug discovery due to the fact of their vast structural diversity with fewer side effects [4]. They have provided important anticancer and antimicrobial leads [5]. For example, paclitaxel, podophyllotoxins, and vinblastine are obtained directly from plants, whereas etoposide, teniposide, irinotecan, and docetaxel are semisynthetic derivatives of these anticancer leads that are being used as anticancer drugs with diverse modes of action. Antimicrobial drugs, such as penicillin, cephalosporins, and fusidic acid, are obtained directly from fungi, while fusidin and tigecycline, which are antibacterial drugs, are semi-synthesized from the leads [5]. Moreover, it has been reported that crude plant extracts contain combinations of many phytochemicals that cause a synergistic effect with available drugs leading to better therapeutic candidates [6].

*Dodonaea viscosa* (*D. viscosa*, Sapindaceae) is an evergreen shrub found in tropical and subtropical regions. Traditionally, it is used for the treatment of various ailments: the stem and leaves are used to treat fever; the leaves and seeds of this plant are used for sore throat; the infusion of roots for cold; stem fumigants for rheumatism [7]. Furthermore, leaves of this plant are useful in relieving itching, acne, and swelling, and it is used as an antispasmodic agent. Roots or leaves decoctions are useful in digestive system disorders, ulcers, constipation, and diarrhea [8]. The plant contains important bioactive compounds such as terpenoids, flavonoids, phenolics, sterols, and sapogenins as reported by previous phytochemical work [9]. The plant extract and its essential oil are reported to exhibit antibacterial, anti-inflammatory, antioxidant, anticancer, antidiabetic, and antiviral activities [10].

*Juniperus procera,* (*J. procera*) belongs to the Cupressaceae family, and it is a medicinal plant located in many countries such as Saudi Arabia, Turkey, and Lebanon. There are more than 65 species of *Juniperus* distributed throughout the world [11]. The plant is endowed with molecules having potential antimicrobial, insecticidal, and anticancer activities. It is used for treating respiratory tract and skin diseases, urinary tract infections, rheumatism, and gall bladder in folk medicines [12,13]. The presence of various important phytochemicals in the plant extracts with diverse pharmacological activities has also been reported [14].

Nanomedicine has received great attention and is becoming the driving force behind a variety of evolutionary and revolutionary changes in drug development. Nanomedicine is used for the treatment of cancer and bacterial infections, as it improves selective targeting of medicine to the tumor cells using passive permeation and active internalization mechanisms [15]. Moreover, nanomedicine decreases drug resistance by cells and increases cytotoxicity against the cancer cells [16]. Furthermore, these nanotextured materials show enhanced antimicrobial activity as well [17]. Inspired by the previous works, which evidently highlight the use of encapsulation, hybridization techniques [18], and cell surface engineering/or coating[19], in this study, we attempted to synthesize plant extracts based on ZnO NRs using coating techniques.

Among different metal oxides, zinc oxide nanoparticles (ZnO NPs) are most effective as anticancer and antimicrobial agents due to the fact of their biocompatibility, solubility, selective delivery, enhanced encapsulation efficiency, and non-toxicity [20]. It is well reported that capped ZnO NPs synthesized from different plant extracts have shown significant cytotoxicity on HepG2, HCT-116, and K562 cancer cells [21,22]. Therefore, in this study, the ZnO nanocomposites of active plant extracts were synthesized to investigate their effect on cancer cells and bacterial strains. 

Stimulated by the numerous pharmacological and phytochemical reports of *D. viscosa* and *J. procera*, analysis of the anticancer and antimicrobial activities of these plants has been carried out. In order to contribute to a better knowledge of these species growing in the Albaha region of Saudi Arabia, the present study reports the anticancer and antimicrobial activities of the extracts of *D. viscosa* and *J. procera* grown in Albaha and their different fractions. The active fractions were further modified to ZnO nanocomposites that were characterized by SEM, TGA, and EDX and evaluated for their biological activities. Even though the advantageous effects of ZnO NPs have captivated substantial attraction in terms of nanomedicine, the possible biological [23] and environmental risks [24] must be taken into consideration. Therefore, in the present study, in addition to evaluating the toxicity of ZnO nanocomposites on cancer cells, we also studied the effect of these composites on normal fibroblast cells. GC-MS of the active fractions from each plant was also carried out to understand the possible phytochemicals responsible for exerting the anticancer and antimicrobial activities by the fractions.

## 2. Experimental

### 2.1. Chemicals

All chemicals and solvents used were of analytical grade. DMSO, Dulbecco’s modified Eagle’s medium (DMEM), Trypsin–EDTA, fetal calf serum (FCS), antibiotic solution, Zn(CH_3_COO)_2_, 2H_2_O salt, and NaOH were procured from Sigma-Aldrich (St. Louis, MO, USA).

### 2.2. Extraction

*Dodonaea viscosa* and *Juniperus procera* were collected from Madarah, Albaha region, Saudi Arabia, and identified by Haider Abd Algadir, Chemotaxonomist, Department of Biology, Albaha University, Saudi Arabia. Both plants were shade-dried and ground into powder. They were Soxhlet extracted with ethanol (95% *v/v*) and completely dried under vacuum at 45 °C in a rotary evaporator to afford *Juniperus* ethanolic extract (JE) and *Dodonaea* ethanolic extract (DE) [25]. Both JE and DE were then fractionated with solvents of increasing polarity using petroleum ether, chloroform, and methanol to give *Juniperus* petroleum ether (JP), *Juniperus* chloroform (JC), *Juniperus* methanolic (JM) fractions from JE and *Dodonaea* petroleum ether (DP), *Dodonaea* chloroform (DC), and *Dodonaea* methanolic (DM) fractions from DE. The extracts (i.e., JE and DE) and all the fractions (i.e., JP, JC, JM, DP, DC, and DM) were in powder form and screened for antimicrobial and anticancer activities.

### 2.3. Gas Chromatography/Mass Spectrometry

The most promising fractions (i.e., JM and DC) from each plant were selected for GC-MS. The fractions (1 μL) were placed onto a Hewlett Packard 7673C Automatic Liquid Sampler (ALS) carousel (Hewlett Packard, Palo Alto, CA, US, where the sample was directly injected into the heated injection port of an Agilent 6890 Gas Chromatograph (GC) Agilent 5973 Mass Selective Detector (MSD) system (Agilent Technologies, Palo Alto, CA, USA). The method used for GC-MS was 50rtndi40.M, and the front inlet parameters were 225 °C and a 50:1 split (2% on-column) direct injection. A MeOH blank was analyzed prior to the sample analysis to identify any residual contaminants present in the system and/or the solvent used to reconstitute the sample. 

### 2.4. Identification of the Components

The compounds present in the fractions were identified by comparison of their mass spectra with those from the Wiley 275 mass spectral database provided by the GC manufacturer and from the literature. The relative percent was computed by dividing the individual compound response by the total outgassing response for the sample and the resultant multiplied by one hundred (100). The Wiley database provides a match quality factor for each search. The match quality indicates how well the unknown spectra match that of a reference library. The components are listed in Table 1 and Table 2.

### 2.5. Synthesis of Pure ZnO Nanorods and Plant-Based Nanocomposites 

ZnO nanorods (ZnO NRs) were primed following an earlier procedure as described elsewhere [77]. Briefly, the ZnO NRs were prepared with a hydrothermal method using Zn(CH_3_COO)_2_·2H_2_O as a precursor. In the present study, 3 g of Zn(CH_3_COO)_2_·2H_2_O was dissolved in 80 mL of deionized water. Then, the aqueous solution was continuously stirred, and sodium hydroxide solution (2 M) was added dropwise until the solution reached a final pH of 12. The final solution was subsequently transferred into a stainless-steel autoclave (100 mL working capacity), and the reaction was kept at 180 °C for 24 h. The reaction mixture was cooled to room temperature. The obtained precipitate was washed with distilled water several times, separated by filtration, and dried in an oven at 80 °C overnight. Pristine ZnO NRs were obtained as an ultimate produce that was in powder form. 

To prepare plant-based ZnO nanocomposites, berries of *J. procera* and the aerial part of *D. viscosa* extracts were used. For the synthesis of JM–ZnO and DC–ZnO nanocomposites, formerly described methods were implemented with sufficient amendments [78,79]. Briefly, approximately 0.5 g of synthesized ZnO NRs were dissolved in methanol in a conical flask under continuous stirring for 3 h. Then, 1 g of JM solution (dissolved in a minimal amount of methanol) was added to the above solution. The ZnO NR solution containing the extract was constantly stirred in a water bath shaker at 60 °C for 48 h. The residual solvents were removed using a rotary evaporator. The DC–ZnO nanocomposites were also prepared following the aforementioned procedure using chloroform as a preparation solvent. The prepared DC–ZnO and JM–ZnO nanocomposites were in powder form and preserved in the dark till further use.

### 2.6. Characterization of Nanocomposites

The X-ray diffraction (XRD) spectra of the synthesized samples (i.e., ZnO NRs and the JM–ZnO and DC–ZnO nanocomposites) were recorded on a Bruker D8 Advance X-ray diffractometer (Bruker, Munich, Germany) with a power of 40 KV and current of 40 mA using CuKα radiation (wavelength: 1.5418 Å), and the X-rays were detected using the fast Lynx Eye one-dimensional detector. The scan was performed using a step size of 0.02 with 2 *θ* range: 10–70 degrees and a time/step: 0.2 s. A scanning electron microscope (SEM; JEOL JSM6700) (Jeol, Ltd., Tokyo, Japan) was used to observe the morphology of the samples. Energy-dispersive X-ray (EDX) spectroscopy attached to the SEM was used to analyze the composition of the ZnO NRs and the JM–ZnO and DC–ZnO nanocomposites. All the samples were coated with Pt before SEM-EDX analyses.

The thermogravimetric analysis (TGA) was carried out using a Mettler Toledo DSC/TGA instrument (Mettler-Toledo, Columbus, OH, USA). All samples were placed into Al_2_O_3_ crucible and then heated from 25 to 800 °C with a heating rate of 10 °C/min under an inert nitrogen stream with a flow rate of 50 mL/min. Weight loss was recorded as a function of temperature, and organic decompositions were determined from the weight loss curve.

### 2.7. Biological Activities 

In the present study, the extracts (i.e., JE and DE) and all the fractions (i.e., JP, JC, JM, DP, DC, and DM) were tested for their antimicrobial and anticancer activities. The most active fractions were taken for the synthesis of the nanocomposites.

#### 2.7.1. Antimicrobial Evaluation

The antimicrobial activity of the extracts (i.e., JE and DE), the fractions (i.e., JP, JC, JM, DP, DC, and DM), and the nanocomposites were screened against *Staphylococcus aureus* (*S. aureus*, ATCC 29213)*, Escherichia coli* (*E. coli*, ATCC 35218), and the fungus *Candida albicans* (*C. albicans*, ATCC 76615). The microorganisms were provided from Dr. Mahmoud Elfaky, (Microbiology Laboratory, King Abdulaziz University Hospital, Jeddah, Saudi Arabia). The bacterial and fungal stock cultures were maintained on Muller–Hinton agar (MHA) plates (Himedia Laboaratory, Mumbai, India), and Sabouraud dextrose agar (SDA) (Himedia Laboaratory, Mumbai, India), respectively. A loopful of overnight microbial cells from the agar plates was inoculated into 5 mL normal saline (85% NaCl), and the turbidity was adjusted to 1–5 × 10^6^ CFU/mL. To check the antimicrobial activity, 100 mg of each extract was dissolved in 1 mL dimethyl sulfoxide (DMSO) (100 mg/mL), vortexed until dissolved completely, then diluted serially to obtain five different concentrations (i.e., 10, 5, 2.5, 1.25, and 0.6125 µg/mL). Similarly, different concentrations of nanocomposites, as mentioned above, were prepared and screened. Preliminary screening of the antibacterial and antifungal activities was conducted using an agar diffusion technique as described previously [80]. Briefly, Petri dishes (90 mm) were filled with 25 mL agar containing 1 mL microbial culture (1 × 10^6^ CFU/mL). The strains were inoculated separately, and 40 µL of each concentration was added to a well of 4 mm in diameter. Dishes were pre-incubated for 2 h at 4 °C to allow pre-diffusion of extracts, then incubated for 24 h at 37 °C. DMSO was used as a negative control, and ciprofloxacin was used as a positive control. Inhibitory activity was defined as the absence of bacterial growth in the area surrounding the holes. The inhibition zone was measured using a caliper.

#### 2.7.2. Anticancer Activity

##### Cell Lines and Culture Medium

The cancer cells, colorectal (HCT-116) and hepatocellular (HepG2), were provided by Thikryat Neamatallah’s Pharmacology Laboratory, Faculty of Pharmacy, King Abdulaziz University, Saudi Arabia. The cells were cultured in DMEM supplemented with 10% (v/v) fetal bovine serum (FBS), 10,000 units/mL penicillin/streptomycin, and 1% (*v/v*) L-glutamine at 37 °C in a humidified 5% CO_2_ incubator.

##### MTT Assay 

The anticancer activity was performed according to a reported method [81]. To assess the cytotoxic effect of the extracts (i.e., JE and DE), fractions (i.e., JP, JC, JM, DP, DC, and DM), and nanocomposites against HCT-116 and HepG2 and cell lines, MTT viability assays were carried out by culturing the cells in a 96-well plate at a density of (3 × 10^3^ cells/well). After 24 h of incubation, the cells were then treated in triplicate with the extracts and fractions at 7 serial dilutions (1000–3.9 μg/mL) for 48 h. Extracts and fractions were dissolved in DMSO with sonication. Appropriate control wells from untreated cells and the vehicle DMSO (0.5%) were prepared at the same time. Then, the medium was removed and replaced with MTT solution (2 mg/mL). The plates were covered with aluminum foil and incubated at 37 °C for 4 h. The purple formazan product was dissolved by adding 200 µL of 100% DMSO. The plates were then incubated for 5 min at 37 °C in a 5% CO_2_ incubator, and the colorimetric signals were measured at 570 nm with a SpectraMax M3 plate reader.

The cytotoxic effect of the extracts and nanocomposites against fibroblast normal cells (3T3) was carried out by an MTT viability assay at 4 serial dilutions (125–15.62 μg/mL) for 48 h. The activity was performed as mentioned above. The colorimetric signals were measured at 490 nm with BioTek plate reader.

## 3. Results

### 3.1. Extraction

The yields of the JE and DE were found to be 13.62% *w/w* and 11.58% *w/w*, respectively. The yields of the fractions of *J. procera* were 29.7% *w/w* (JP), 37.4% *w/w* (JC), and 32.9% *w/w* (JM), while the fraction yields from *D. viscosa* were 31.2% *w/w* (DP), 29.1% *w/w* (DC), and 39.7% *w/w* (DM).

### 3.2. GC-MS Analysis

The two most active fractions (i.e., JM and DC) were analyzed by GC-MS to determine the chemical constituents present in each fraction that imparted antimicrobial and anticancer activities to these fractions (see Figures S1 and S2 in supplementary materials file). From the JM fractions, the top 30 compounds were integrated and identified. These compounds were limonene, umbellulone, hydroxyl methyl pyranone, hydroxymethylfurfural, cedrol, bisabolene, dehydroabietic acid, 4-phenoxy phenol, sclareol, pimarinal, androstadienone, totarol, tataradiol, kaurenoic acid and isopimara-7(8), and 15-diene-19-oic acid (Table 1). The DC fractions showed the presence of flavonoids, phenolics, terpenoids and cleoradanes. The flavonoids included quercetin, eriodictyol 7-methyl ether, kaempferol, vetulin, centauridin, naringenin 7-O-methyl ether, and 6-hydroxykaempferol 3,6,7-trimethyl ether. The terpenoids present were betulin, stigmasterol, and dodonaeaside A and B, while cleoradanes were 13,14 dihydroxy-15,16 dimethoxy-(-)-6α-hydroxy-5α; 8α, 9α, 10α-cleroda-3-en-18-oic acid; (–)-6α-hydroxy-5α, 8α, 9α, 10α-cleroda-3,13-dien-16,15-olid-18-oic acid. The phenolics were hautriwaic acid, 2-methoxy-4-vinylphenol, phytol, and γ-tocopherol. The other phytochemicals were costunilide and 5-(3-buten-1-Ynyl)-2,2’-bithienyl. The structures with their reported antimicrobial and anticancer activities are presented in Table 2. 

Compared to the literature on *D. viscosa*, dodonaeaside A and B have been reported from the ethanolic extract of *D. viscosa* leaves from the Madagascar Dry Forest by Cao et al. [71]. Stigmasterol, tocopherol, 5-(3-Buten-1-Ynyl)-2,2’-Bithienyl, costulinide, phytol, 2-methoxy-4-vinyl phenol were obtained from the methanolic leaves extract of *D. viscosa* grown in India by Ansarali et al. [82]. Flavonoids such as eriodyctiol, velutin, centauridin, 7-O-methylnaringenin, naringenin 7-O-methyl ether, kaempferol, 6-hydroxykaempferol-3,6,7,4’-tetramethyl ether, quercetin, and isorhamnetic were reported from the ethanolic and acetone extract of leaves [83,84]. Hautriwaic acid was obtained from the methanolic leaves extract by Al-Bimani et al. from Oman [65]. Cleoradanes; 13,14 dihydroxy-15,16 dimethoxy-(-)-6α-hydroxy-5α, 8α, 9α, 10α-cleroda-3-en-18-oic acid; (–)-6α-hydroxy-5α, 8α, 9α, 10α-cleroda-3,13-dien-16,15-olid-18-oic acid; 1-L-O-methyl-2-acetyl-3-p-cis-coumaryl-myo-inositol have been reported from the leaves of ethanolic extract of *D. viscosa* found in Egypt [59].

*Juniperus* sp. literature revealed the presence of limonene, cedrene, camphene, caryophyllene, muurolene, humulene, calocorene, cedrol, valencene, neophytadiene, and phenoxy phenol in the essential oils of J. *foetidissima* leaves and berries grown in R. Macedonia [49]. Dehydroabietic acid and totarol occur widely in several Juniperus species including *J. procera* [52]. 

### 3.3. Characterization of Nanocomposites

**Figure 1:**
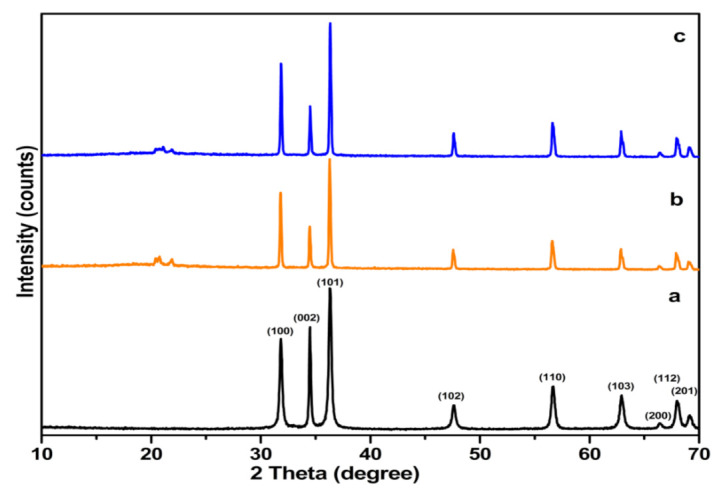
XRD of (**a**) pure ZnO NRs and (**b**) DC–ZnO and (**c**) JM–ZnO nanocomposites.

Figure 1 shows the XRD spectra of pure ZnO NRs and nanocomposites of ZnO. As implied in Figure 1a, in the spectrum of ZnO NRs, all diffraction peaks were similar to standard hexagonal wurtzite ZnO [77,85] and showed strong and sharp peaks indicating a superior crystallized morphology of the prepared nanorod samples. No obvious additional peaks of impurities were observed in the obtained spectrum of ZnO NRs. After modification of ZnO nanoparticles with *J. procera methanol* (JM) and *D. viscosa* chloroform (DC) fractions, several other phases apart from ZnO were observed. These peaks were found to be comparatively less intense, and also two small additional peaks appeared at 2θ = 20.5° and 21.8°, respectively, which may be attributed to the presence of unknown biomolecules in the plant extracts that capped ZnO NRs (Figure 1b,c). 

**Figure 2:**
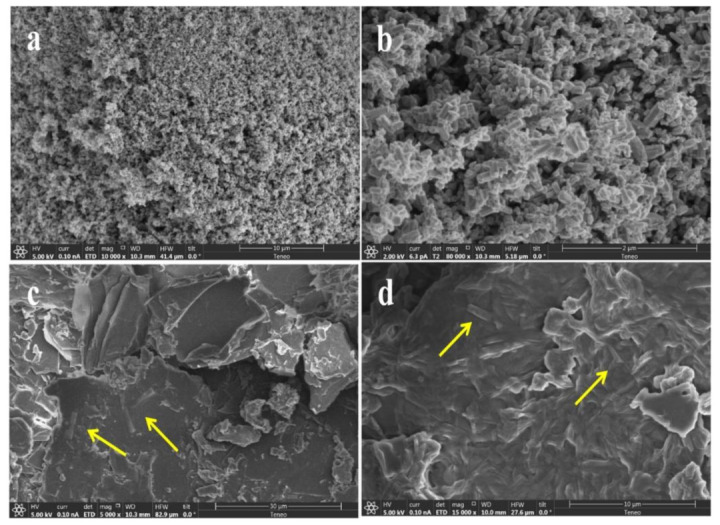
SEM of (**a**,**b**) pure ZnO NRs at low and high magnifications and (**c**) JM–ZnO and (**d**) DC–ZnO nanocomposites. Yellow arrows show the representative coated ZnO NRs.

Therefore, these results clearly indicate that the ZnO and plant extract nanocomposites were synthesized. The diffractograms of the DC–ZnO NRs [86] as well as the JM–ZnO NRs [87] confirmed the presence of both ZnO and plant extracts; however, no sharp peaks originating from respective plant materials were present due to the amorphous nature of organic extracts. Figure 2 shows SEM morphological images of the prepared ZnO and the DC–ZnO and JM–ZnO nanocomposites. Uniformly distributed nanorods can be observed for the pure ZnO sample (Figure 2a). The average size of the nanorods was calculated to be approximately 80 nm in width and 200 nm in length. Whereas in the case of the DC–ZnO and JM–ZnO composites, the organic fractions of *J. procera* and *D. viscosa* showed smooth surfaces with ZnO NRs embedded in the surface (Figure 2b,c). 

**Figure 3:**
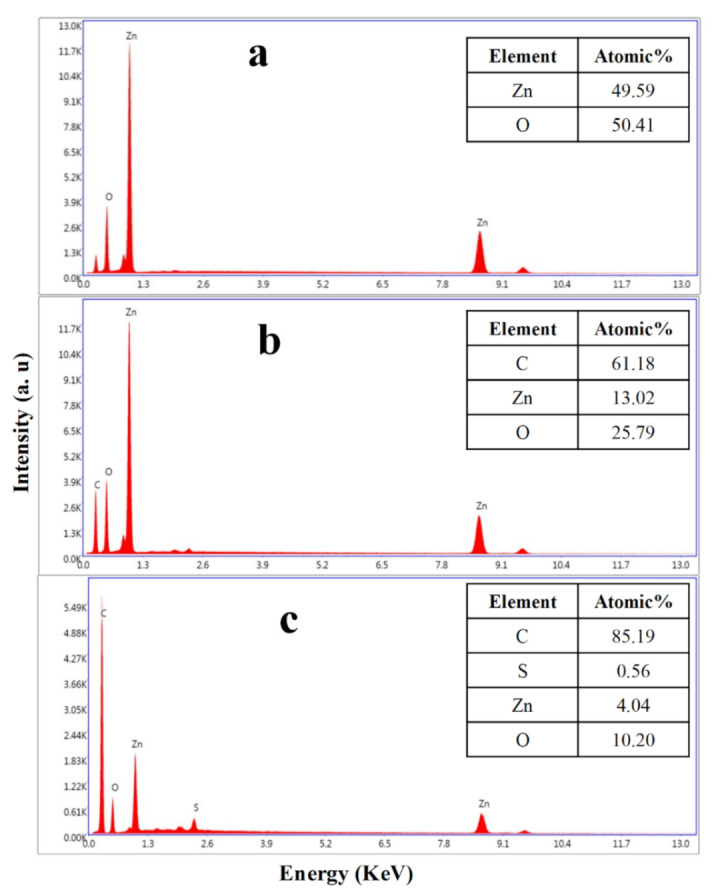
Elemental compositions (EDX analysis) of (**a**) pure ZnO NRs and (**b**) JM–ZnO and (**c**) DC–ZnO nanocomposites.

The SEM, again, confirmed the formation of the DC–ZnO and JM–ZnO nanocomposites. The EDX spectrum of the prepared ZnO NRs is shown in Figure 3, displaying that the composition was Zn and O only, without any impurities. The EDX spectrum of ZnO NRs revealed the absolute elimination of unwanted organics and the formation of metal oxide species (Figure 3a). The EDX of JM–ZnO composites revealed the presence of principally C and O in addition to Zn (Figure 3b). Whereas, in the case of the DC–ZnO nanocomposite, the presence of C, Zn, O, and a small percentage of S was detected due to the presence of minerals and trace elements in the DC extract [88] or the presence of the organic biomolecule, 5-(3-buten-1-ynyl)-2,2’-bithienyl, which has been reported from the extract of leaves of this plant [69] (Figure 3c). The increase in the intensities of C and O molecules in both the nanocomposites further confirmed the formation of DC–ZnO and JM–ZnO NRs. 

**Figure 4:**
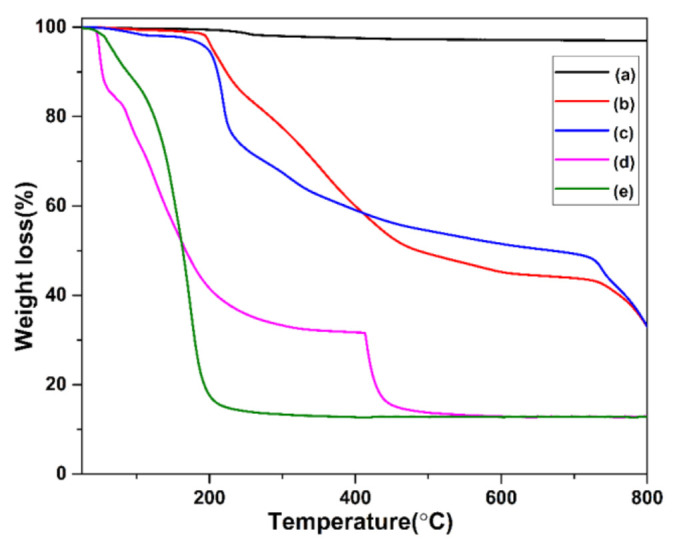
Thermal analysis of (**a**) pure ZnO NRs; (**b**) DC–ZnO and (**c**) JM–ZnO nanocomposites; (**d**) JM; (**e**) DC.

The thermogravimetric analysis (TGA) of the ZnO NRs and the DC–ZnO and JM–ZnO nanocomposites are shown in Figure 4. 

In the case of the ZnO NRs’ thermogram, a total weight loss of approximately 3% was found in the temperature range from 25 to 800 °C due to the removal of the moisture and adsorbed structural water (Figure 4a). These results are in line with the results of the TG analysis of ZnO nanoparticles reported by other authors in a similar temperature range [89]. 

In the thermograms of the DC–ZnO NRs and JM–ZnO NRs, a two-stage thermal degradation can be seen. In both the cases, in the first stage, a 1% and 3% weight loss was observed for the DC–ZnO and JM–ZnO nanocomposites [87], respectively, between a temperature range of 25 and 190 °C, attributed to the loss of moisture and adsorbed structural water. In the second stage, a weight loss of 64% was observed in the JM–ZnO NRs, which might be due to the decomposition of molecular organic compounds such as phenolics, 4-phenoxy phenol, sclareol, totarol, kaurenoic acid, dehydroabietic acid, totaradiol, and isopimara-7(8),15-dien-19-oic acid in the JP extract as reported in Table 2. On the other hand, a 62% weight loss was calculated in the DC–ZnO NRs between the temperature range of 180 and 800 °C, owing to degradation of organic compounds such as quercetin, velutin, eriodictyol, rhamnetin, costunilide, dodonaeaside A and B, and cleoradanes in the DV extract as reported in Table 2 (Figure 4b,c). The thermal spectra of the JM and DC extracts depicted an ~87.5% degradation until 800 °C due to the disintegration of organic compounds as aforementioned in the Table 1 and Table 2, respectively (Figure 4d,e). The major weight loss (~80%) was observed until 200 °C in DC extract, whereas JM demonstrated two-stage decomposition. The first stage of weight loss (~68.5%) was observed until 400 °C, and in the second stage, a weight loss of ~86% was observed until 500 °C. 

Consequently, a total weight loss of approximately 65% was noticed for the DC–ZnO and JM–ZnO NRs which once again established the successful formation of ZnO and plant extract (JM and DC) nanocomposites.

### 3.4. Antimicrobial Activity 

**Figure 5:**
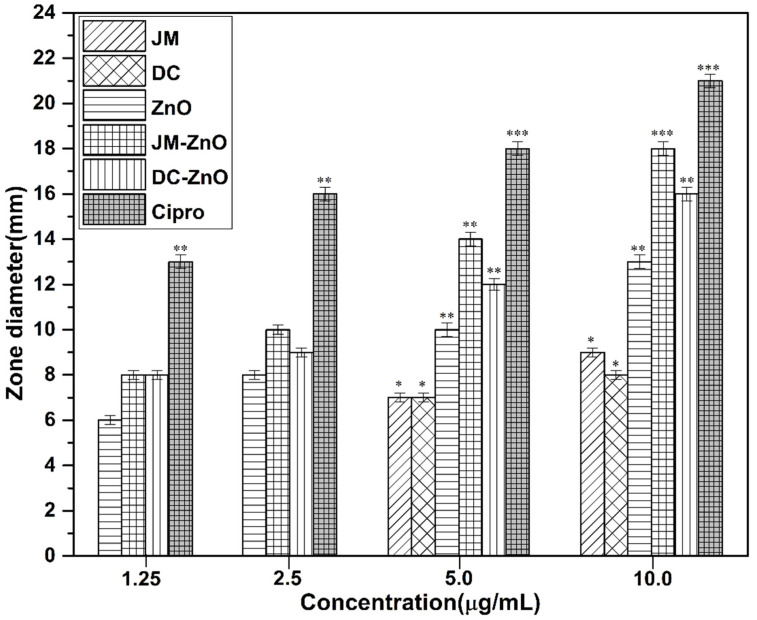
Antimicrobial susceptibility pattern of *Staphylococcus aureus* with fractions and nanocomposites (10–1.25 µg/mL). Ciprofloxacin was used as a reference drug (positive control). Data represent the mean values ± standard deviation of three replicates. Statistical analysis was performed by Dunnett’s test. * *p* < 0.05, ** *p* < 0.01, and *** *p* < 0.005 vs. the control.

In an in vitro antimicrobial bioassay, the eleven extracts were evaluated by agar diffusion assay using different concentrations on *S. aureus* (ATCC 29213)*, E. coli* (ATCC 35218), and *C. albicans* (ATCC 76615). It was observed that the methanolic fraction (JM) from *J. procera* and the chloroform fraction (DC) from *D. viscosa* were the most promising fractions with an MIC of 5 μg/mL against *S. aureus*. The other fractions and extracts displayed moderate activity. The most promising fractions (JM, DC) were chosen for synthesis of the nanocomposites. The synthesized ZnO NRs and nanocomposites of DC (DC–ZnO NRs) and JM (JM–ZnO NRs) displayed excellent antimicrobial activity against Gram-positive *S. aureus* with MICs of 2.5 and 1.25 μg/mL, respectively. The results are shown in Figure 5. But moderate antibacterial action was observed with DC–ZnO and JM–ZnO nanocomposites against selected Gram-negative bacteria, *E. coli.* However, no prominent antifungal activity was observed with the extracts and fractions as well as the nanocomposites in the present study.

The antimicrobial activity of the extracts and fractions might be attributed to the presence of antimicrobial compounds: santin; quercetin; hautriwaic acid; 5-(3-buten-1-ynyl)-2,2’-bithienyl; 13,14 dihydroxy-15,16-dimethoxy-(-)-6α-hydroxy-5α, 8α, 9α, 10α-cleroda-3-en-18-oic acid; 1-L-O-methyl-2-acetyl-3-p-cis-coumaryl-myo-inositol in the DV extract (Table 2). In the JM fraction, the antimicrobial compounds limonene, calacorene, umbellulone, pimarinal, and totaradiol may be imparting antimicrobial activity. The enhanced activity of the ZnO NRs and nanocomposites was attributed to the morphology and high surface area [90] as well as a synergistic effect. Although the pristine fractions (JM, DC) displayed the same activity at low concentration (5 μg), the antimicrobial effect varied in the respective nanocomposites, as both fractions contain different metabolites (Table 1 and Table 2). The difference in activity was attributed due to the difference in the chemical components of the fractions and their synergism with ZnO NRs. The chemical components of DC–ZnO and JM–ZnO NRs might have interacted with the bacterial outer cell wall initially and then diffused into the inner cell wall, causing disorganization and leakage by disruption of the inner cell’s content and cell deformation. In the present study, it was also noted that *S. aureus* was more sensitive to the tested samples compared to the other tested strains. The greater sensitivity of *S. aureus* has been credited to the difference in the cell organization of the strains [90,91]. Typically, the configuration of the microorganisms is essentially responsible for the differences in their sensitivity.

### 3.5. Anticancer Activity

**Figure 6:**
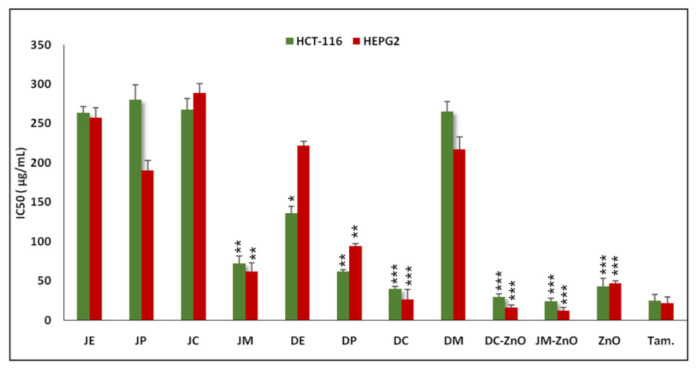
Anticancer activity of different extracts and nanocomposites against the HCT-116 and HepG2 cell lines. Data represent the mean values ± standard deviation of three independent experiments performed in triplicate. Tamoxifen was used as the reference drug (i.e., positive control). Statistical analysis was performed by Dunnett’s test. * *p* < 0.05, ** *p* < 0.01, and *** *p* < 0.005 vs. the control.

The anticancer activity of the extracts, its fractions, and the nanocomposites were performed on two cancer cell lines: HepG2 (liver cancer cells) and HCT-116 (colorectal cell lines). Tamoxifen was used as the standard anticancer agent. Firstly, the extracts (JE, DE) and their different fractions (JP, JC, JM, DP, DC, DM) were evaluated for cytotoxicity on the two cell lines. It was observed that the methanolic fraction (JM) from *J. procera* was the most active fraction with an IC_50_ of 62.98 ± 9.8 (HepG2) and 72 ± 11 μg/mL (HCT-116), whereas the chloroform fraction (DC) was potent from *D. viscosa* with an IC_50_ 26.4 ± 3.3 (HepG2) and 39.8 ± 13 μg/mL (HCT-116). The remaining fractions and the ethanolic extract displayed moderate cell viability against these cell lines (Figure 6). Therefore, we selected active fractions (JM, DC) from each plant for the preparation of their nanocomposites with zinc to see whether the synthesized nanocomposites would enhance further cytotoxicity. Interestingly, we found that the nanocomposite DC–ZnO and JM–ZnO NRs enhanced cytotoxicity in the HepG2 and HCT-116 cell lines. The nanocomposite DC–ZnO NRs exhibited cytotoxicity with an IC_50_ of 16.4 ± 4 (HepG2) and 29.07± 2.7 μg/mL (HCT-116), while JM–ZnO NRs had an IC_50_ of 12.2 ± 10.27 (HepG2) and 24.1 ± 3.0 μg/mL (HCT-116). 

Although the diverse applications of nanomaterials have resulted in the exponential progression of nano-textured materials over recent years. However, in spite of their wide range of applications, little information is available about their consequences on human health and the environment [92]. There are some reports in the literature that have clearly indicated that accidental exposure to nanomaterials via inhalation, skin contact, or absorption through the gastrointestinal route can pose pronounced threat to humans [93]. 

**Figure 7:**
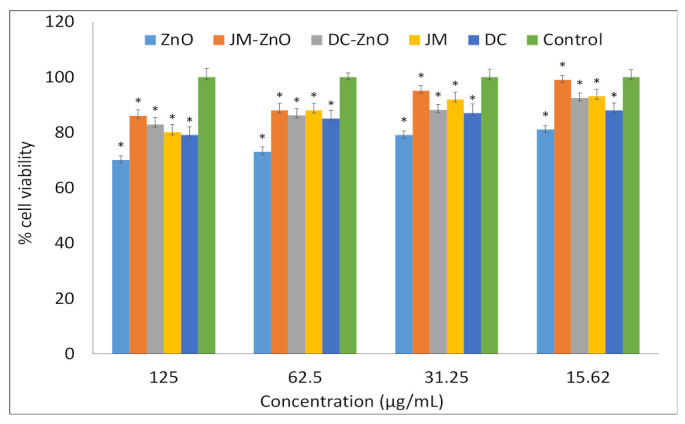
Anticancer activity of different extracts and nanocomposites on normal fibroblast (3T3) cells. Data represent the mean values ± standard deviation of three independent experiments performed in triplicate. Statistical analysis was performed by Dunnett’s test. * *p* < 0.05 vs. the control.

To assess the safety of these extracts and nanocomposites on normal cells, the cytotoxicity of these extracts and nanocomposites were performed on normal fibroblast (3T3) cells. As shown in Figure 7, the tested extracts and nanocomposites did not exhibit significant toxicity or there was no significant difference in cell viability with respect to the untreated control cells. As a result, these extracts exclusively had cytotoxic effects on malignant cells and no harmful effect on normal cells. 

It has been reported that sclareol, a plant diterpene, inhibits the growth of osteosarcoma tumor cells MG63 (IC_50_ of 65.2 μM); inhibits cell proliferation in leukemic, breast, and HeLa cancer cells; induces apoptosis via regulation of the caveolin-1 (Cav-1) protein P13K, STAT5, and NF-kB pathways [94]. Totarol, a diterpenoid exerts anticancer effects on gastric cancer cells, SGC-7901, by induction of apoptosis, cell cycle arrests, and suppression of cancer cell migration [51]. Phenoxy phenol downregulates the expression of α-tubulin and exerts inhibitory effects on hepatocellular carcinoma cells [41]. Isopimara-7,15-dien-19-oic acid has been reported to cause cell death in HeLa by inducing apoptosis through regulation of BCL-2 and FAS genes [50]. Kaurenoic acid, an ent-kaurene-type diterpene exhibits antiproliferative effect in breast, gastric, 293T, HeLa, PANC-1, and cervical cell lines by inducing apoptosis in tumor cells through regulation of the c-FLIP, caspase-3, caspase-8, and miR-2 pathways [95] The presence of these phytochemicals in JM fractions as observed in GC-MS are responsible for the anticancer effect of *J*. *procera*.

*D. viscosa* is a flavonoid-rich plant. It is well known that flavonoids exert anticancer effects through many mechanisms. Naringenin has been reported to exert anticancer effects in breast (MDA-MB-231), hepatocellular (HepG2), mammary tumor (E0771), and prostate (PC3, LNCaP) cancer cells by arresting the cell cycle and inducing apoptosis. It also causes suppression of melanoma SK-MEL-28 cells by inhibiting ERK1/2 and JNK MAPKs’ phosphorylation [96]. Eriodictyol exerts antiproliferation and anti-metastasis in brain tumor cells by blocking of signaling pathways (NF-kB, PI3K) and induction of apoptosis [97]. Penduletin -4’-methyl ether has been reported to be an aromatase inhibitor with an IC_50_ of 1.0 μM [62]. Quercetin, a polyphenolic flavonoid, is useful in cancer prevention as it prevents the various types of cancers. It causes suppression of p38MAPK, the MAPKs pathway in CT26 cells, blocking of the NF-κB pathway, activation of caspase-3, inhibition of EGFR, and increases in the expression of miR-146a [63]. In addition to flavonoids, this plant also contains anticancer terpenoids and phenolics. Stigmasterol, a terpenoid, inhibits cell proliferation in SGC-7901and MGC-803 cells and induces apoptosis and autophagy by blocking Akt/mTOR signaling pathway [75]. Dodonaeaside A and B, triterpenoids, exerted promising anticancer activity with IC_50_ values of 0.79 and 0.70 μM, respectively, towards the human ovarian (A2780) cancer cell line [76]. Costunolide, a sesquiterpene lactone, exerts anticancer activities by inducing apoptosis, inhibiting cell proliferation and metastasis as well as angiogenesis [71]. All these compounds reported in *D. viscosa* could be responsible for the significant anticancer activity exhibited by DC fractions.

Nanomaterials are expected to revolutionize cancer diagnosis and therapy [98]. In this regard, zinc oxide nanomaterials have proved to be one of the most effective candidates in nanomedicine. In this study, the enhanced cytotoxicity of ZnO NRs against selected cancer cells is ascribed to the release of dissolved zinc ions and the induction of the reactive oxygen species, which leads to apoptosis of cancerous cells [20,99]. Moreover, the higher in vitro anticancer activity of JM–ZnO and DC–ZnO nanocomposites over the blank nanorods is attributed to the capping of the abovementioned bioactive compounds (Table 1 and Table 2) present in the extracts. The synergistic effect between nanorods and active extracts has resulted in potent anticancer activity of the JM–ZnO and DC–ZnO nanocomposites against the HCT-116 and HepG2 cancer cell lines.

## 4. Conclusions

It can be concluded that JM–ZnO and DC–ZnO nanocomposites were successfully synthesized using native Albaha resources and also characterized by various techniques. *D. viscosa* and *J. procera* and their JM–ZnO and DC–ZnO nanocomposites possessed anticancer and antimicrobial activities that might be attributed to the presence of important bioactive compounds present in these plants. Additionally, the safety of these extracts and nanocomposites was assessed on normal fibroblast cells and had non-cytotoxic effects on these cells. The GC-MS analysis of fractions directed the tentative identification of possible phytochemicals that are responsible for these activities. Future studies will be focused on the isolation of these bioactive molecules, which can be utilized in the preparation of nano-drugs with appropriate consideration of safety procedures. 

## Figures and Tables

**Table 1:** Chemical constituents of the JM fraction seen by GC-MS.

Name	Structure	Activities	References
Limonene	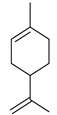	Anticancer Antimicrobial	[26,27]
Umbellulone	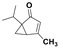	Antimicrobial	[28]
Dihydro dihydroxy methyl pyranone	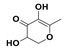	Anticancer	[29]
Hydroxymethylfurfural	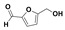	Anticancer	[30]
Camphene	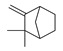	Anticancer Antimicrobial	[31,32]
Cedrene	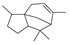	Anticancer Antimicrobial	[33]
Caryophyllene	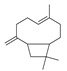	Anticancer Antimicrobial	[34]
Muurolene	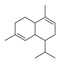	Anticancer Antimicrobial	[35]
Humulene	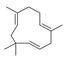	Anticancer Antimicrobial	[36,37]
Calacorene	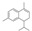	Antimicrobial	[38]
Cedrol	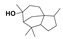	Anticancer	[39]
Valencene	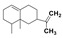	Anticancer Antimicrobial	[40]
Neophytadiene	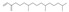	Anticancer	[41]
Bisabolene	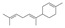	Anticancer Antimicrobial	[42,43]
Dehydroabietic acid	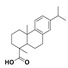	Anticancer Antimicrobial	[44]
Methyl linoleate	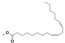	Antimicrobial	[45]
4-Phenoxy phenol	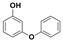	Anticancer	[46]
Sclareol	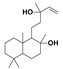	Anticancer Antimicrobial	[47,48]
Pimarinal	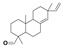	Antimicrobial	[49]
Androstadienone	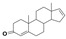	Anticancer	[50]
Totarol	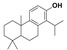	Anticancer Antimicrobial	[51,52]
Totaradiol	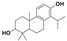	Antimicrobial	[53]
Kaurenoic acid	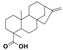	Anticancer Antimicrobial	[54]
Isopimara-7(8),15-dien-19-oic acid	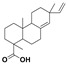	Anticancer	[55]

**Table 2:** Chemical compounds from *D. viscosa* with anticancer and antimicrobial activities.

Name	Structure	Activity	References
4H-1-Benzopyran-4-one, 2-(3,4-dihydroxyphenyl)-2,3-dihydro-5-hydroxy-7-methoxy (Eriodictyol 7-methyl ether; Sternbin; Sterubin)	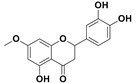	Anticancer	[56]
5-Hydroxy-2-(4-hydroxy-3-methoxyphenyl)-7-methoxy-4H-1-benzopyran-4-one (Velutin)	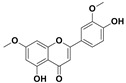	Anticancer	[57]
5,7-Dihydroxy-3,6-dimethox y-2-(4-methoxyph enyl)-4H-1-benzopyran-4-one (Centauridin; Santin)	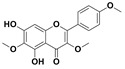	Anticancer Antimicrobial	[58,59]
2,3-Dihydro-5-hydroxy-7-methoxy-2-(4-methoxyphen yl)-4H-1-benzopyran-4-one (7,4’-O-Dimethylnaringenin; Naringenin 4’,7-dimethyl ether)	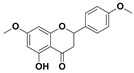	Anticancer Antimicrobial	[60]
2,3-Dihydro-5-hydroxy-2-(4-hydroxyphenyl)-7-methoxy-4H-1-benzopyran-4-one (7-O-Methylnaringenin; Naringenin 7-O-methyl ether)	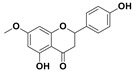	Anticancer Antimicrobial	[55]
3,5,7-Trihydroxy-2-(4-hydro xy phenyl)-4H-1-benzopyran-4-one (Kaempferol)	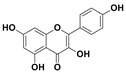	Anticancer	[61]
5-Hydroxy-3,6,7,4’-tetramethoxyflavone (6-Hydroxykaempferol-3,6,7,4’-tetramethyl ether; Penduletin-4’-methyl ether)	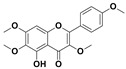	Anticancer Antimicrobial	[59,62]
4’,5-Dihydroxy-3,6,7-trimethoxyflavone (6-Hydroxykaempferol 3,6,7-trimethyl ether)	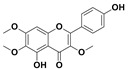	Anticancer Antimicrobial	[57,59]
3, 5, 7, 3’,4’-pentahydroxy flavones (Quercetin)	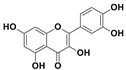	Anticancer Antimicrobial	[63]
Isorhamnetin	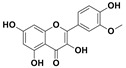	Anticancer, Antimicrobial	[64]
Hautriwaic acid	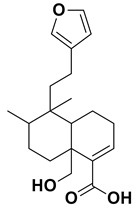	Antimicrobial	[65]
2-Methoxy-4-vinylphenol	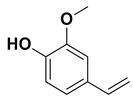	Antimicrobial	[66]
Betulin	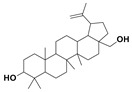	Antimicrobial Anticancer	[67,68]
Phytol	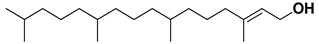	Antimicrobial	[69]
Costunilide	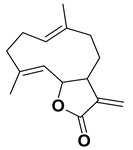	Antimicrobial Anticancer	[70,71]
5-(3-Buten-1-Ynyl)-2,2’-bithienyl	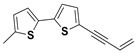	Antimicrobial	[72]
Gamma-tocopherol	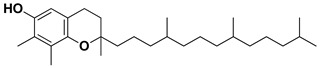	Anticancer Antimicrobial	[73]
Stigmasterol	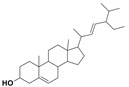	Antimicrobial Anticancer	[74,75]
Dodonaeaside A and B	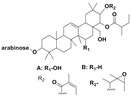	Anticancer	[76]
13,14 dihydroxy-15,16 dimethoxy-(-)-6 α-hydroxy-5α, 8α, 9α, 10α-cleroda-3-en-18-oic acid	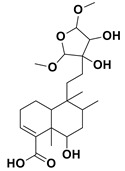	Antimicrobial	[59]
(–)-6α-Hydroxy-5α, 8α, 9α, 10α-cleroda-3,13-dien-16,15- olid-18-oic acid	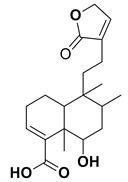	Antimicrobial	[59]
1-L-O-Methyl-2-acetyl-3-p-cis-coumaryl-myo-inositol, 382	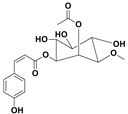	Antimicrobial	[59]

## Data Availability

Not applicable.

## Supplementary Materials

The following are available online at https://www.mdpi.com/article/10.3390/nano12040664/s1, Figure S1: GC-MS chromatogram of *D. viscosa* chloroform fraction; Figure S2: GC-MS chromatogram of *J. procera* methanolic fraction.

## References

[1] Almalki A.S., Nazreen S., Malebari A.M., Ali N.M., Elhenawy A.A., Alghamdi A.A., Ahmad A., Alfaifi S.Y., Alsharif M.A., Alam M.M. (2021). Synthesis and Biological Evaluation of 1,2,3-Triazole Tethered Thymol-1,3,4-Oxadiazole Derivatives as Anticancer and Antimicrobial Agents. Pharmaceuticals.

[2] Rostom S.A., Badr M.H., Abd H.A., El Razik Ashour H.M., Abdel Wahab A.E. (2011). Synthesis of some pyrazolines and pyrimidines derived from polymethoxy chalcones as anticancer and antimicrobial agents. Arch. Pharm..

[3] Rajanarendar E., Reddy M.N., Krishna S.R., Reddy K.G., Reddy Y., Rajam M. (2012). Design, synthesis, in vitro antimicrobial and anticancer activity of novel methylenebis-isoxazolo [4,5-b] azepines derivatives. Eur. J. Med. Chem..

[4] Alam M.M., Malebari A.M., Syed N., Neamatallah T., Almalki A.S., Elhenawy A.A., Obaid R.J., Alsharif M.A. (2021). Design, synthesis and molecular docking studies of thymol based 1,2,3-triazole hybrids as thymidylate synthase inhibitors and apoptosis inducers against breast cancer cells. Bioorg. Med. Chem..

[5] Abdel-Razek A.S., El-Naggar M.E., Allam A., Morsy O.M., Othman S.I. (2020). Microbial natural products in drug discovery. Processes.

[6] Neergheen V.S., Bahorun T., Taylor E.W., Jen L.-S., Aruoma O.I. (2010). Targeting specific cell signaling transduction pathways by dietary and medicinal phytochemicals in cancer chemoprevention. Toxicology.

[7] Rani M.S., Pippalla R.S., Mohan K. (2009). *Dodonaea viscosa* Linn—An overview. Asian J. Pharm. Res. Health Care.

[8] Shanmugavasan A., Ramachandran T. (2011). Investigation of the extraction process and phytochemical composition of preparations of *Dodonaea viscosa* (L.) Jacq. J. Ethnopharmacol..

[9] Marvilliers A., Illien B., Gros E., Sorres J., Kashman Y., Thomas H., Smadja J., Gauvin-Bialecki A. (2020). Modified Clerodanes from the Essential Oil of Dodonea viscosa Leaves. Molecules.

[10] Lawal D., Yunusa I. (2013). *Dodonea Viscosa* Linn: Its medicinal, pharmacological and phytochemical properties. Int. J. Innov. Appl. Stud..

[11] Abdelghany T., Hassan M.M., El-Naggar M.A., Abd El-Mongy M. (2020). GC/MS analysis of *Juniperus procera* extract and its activity with silver nanoparticles against Aspergillus flavus growth and aflatoxins production. Biotechnol. Rep..

[12] Öztürk M., Tümen I., Uǧur A., Aydoǧmuş-Öztürk F., Topçu G. (2011). Evaluation of fruit extracts of six Turkish Juniperus species for their antioxidant, anticholinesterase and antimicrobial activities. J. Sci. Food Agric..

[13] Ibrahim E.H., Kilany M., Ghramh H.A., Khan K.A., Islam S.U. (2019). Cellular proliferation/cytotoxicity and antimicrobial potentials of green synthesized silver nanoparticles (AgNPs) using *Juniperus procera*. Saudi J. Biol. Sci..

[14] Bakri M.M., El-Naggar M.A., Helmy E., Ashoor M.S., Ghany T.A. (2020). Efficacy of *Juniperus procera* constituents with silver nanoparticles against Aspergillus fumigatus and Fusarium chlamydosporum. BioNanoScience.

[15] Pillai G., Mohapatra S.S., Ranjan S., Dasgupta N., Mishra R.K., Thomas S. (2019). Nanotechnology toward Treating Cancer: A Comprehensive Review. Nanoscience and Nanotechnology in Drug Delivery Micro and Nano Technologies.

[16] Anjum S., Hashim M., Malik S.A., Khan M., Lorenzo J.M., Abbasi B.H., Hano C. (2021). Recent Advances in Zinc Oxide Nanoparticles (ZnO NPs) for Cancer Diagnosis, Target Drug Delivery, and Treatment. Cancers.

[17] Wang L., Hu C., Shao L. (2017). The antimicrobial activity of nanoparticles: Present situation and prospects for the future. Int. J. Nanomed..

[18] Geng W., Wang L., Jiang N., Cao J., Xiao Y.-X., Wei H., Yetisen A.K., Yang X.Y., Su B.-L. (2018). Single cells in nanoshells for the functionalization of living cells. Nanoscale.

[19] Geng W., Jiang N., Qing G.-Y., Liu X., Wang L., Busscher H.J., Tian G., Sun T., Wang L.-Y., Montelongo Y. (2019). Click reaction for reversible encapsulation of single yeast cells. ACS Nano.

[20] Bisht G., Rayamajhi S. (2016). ZnO nanoparticles: A promising anticancer agent. Nanobiomedicine.

[21] Abbasi B.A., Iqbal J., Ahmad R., Zia L., Kanwal S., Mahmood T., Wang C., Chen J.-T. (2020). Bioactivities of Geranium wallichianum leaf extracts conjugated with zinc oxide nanoparticles. Biomolecules.

[22] Ahlam A.A., Shaniba V., Jayasree P., Kumar P.M. (2021). Spondias pinnata (Lf) Kurz Leaf Extract Derived Zinc Oxide Nanoparticles Induce Dual Modes of Apoptotic-Necrotic Death in HCT 116 and K562 Cells. Biol. Trace Elem. Res..

[23] Sahu D., Kannan G., Vijayaraghavan R., Anand T., Khanum F. (2013). Nanosized zinc oxide induces toxicity in human lung cells. Int. Sch. Res. Not..

[24] Czyżowska A., Barbasz A. (2020). A review: Zinc oxide nanoparticles–Friends or enemies?. Int. J. Environ. Health Res..

[25] Nazreen S., Mahboob Alam M., Hamid H., Ali M., Sarwar Alam M. (2020). Chemical constituents with antimicrobial and antioxidant activity from the aerial parts of Callistemon lanceolatus (Sm.) Sweet. Nat. Prod. Res..

[26] Yu X., Lin H., Wang Y., Lv W., Zhang S., Qian Y., Deng X., Feng N., Yu H., Qian B. (2018). D-limonene exhibits antitumor activity by inducing autophagy and apoptosis in lung cancer. OncoTargets Ther..

[27] Espina L., Gelaw T.K., de Lamo-Castellvi S., Pagán R., Garcia-Gonzalo D. (2013). Mechanism of bacterial inactivation by (+)-limonene and its potential use in food preservation combined processes. PloS ONE.

[28] Teke G.N., Elisée K.N., Roger K.J. (2013). Chemical composition, antimicrobial properties and toxicity evaluation of the essential oil of Cupressus lusitanica Mill. leaves from Cameroon. BMC Complementary Altern. Med..

[29] Ban J.O., Hwang I.H., Kim T.M., Hwang B.Y., Lee U.S., Jeong H.-S., Yoon Y.D., Kim D.J., Hong J.T. (2007). Anti-proliferate and pro-apoptotic effects of 2, 3-dihydro-3, 5-dihydroxy-6-methyl-4H-pyranone through inactivation of NF-κB in human colon cancer cells. Arch. Pharm. Res..

[30] Zhao L., Chen J., Su J., Li L., Hu S., Li B., Zhang X., Xu Z., Chen T. (2013). In vitro antioxidant and antiproliferative activities of 5-hydroxymethylfurfural. J. Agric. Food Chem..

[31] Girola N., Figueiredo C.R., Farias C.F., Azevedo R.A., Ferreira A.K., Teixeira S.F., Capello T.M., Martins E.G., Matsuo A.L., Travassos L.R. (2015). Camphene isolated from essential oil of Piper cernuum (Piperaceae) induces intrinsic apoptosis in melanoma cells and displays antitumor activity in vivo. Biochem. Biophys. Res. Commun..

[32] de Freitas B.C., Queiroz P.A., Baldin V.P., do Amaral P.H., Rodrigues L.L., Vandresen F., Caleffi-Ferracioli K.R., del Scodro R.B., Cardoso R.F., Siqueira V.L. (2020). (-)-Camphene-based derivatives as potential antibacterial agents against Staphylococcus aureus and *Enterococcus* spp.. Future Microbiol..

[33] Su Y.-C., Hsu K.-P., Wang E.I.-C., Ho C.-L. (2012). Composition, anticancer, and antimicrobial activities in vitro of the heartwood essential oil of Cunninghamia lanceolata var. konishii from Taiwan. Nat. Prod. Commun..

[34] Dahham S.S., Tabana Y.M., Iqbal M.A., Ahamed M.B., Ezzat M.O., Majid A.S., Majid A.M. (2015). The anticancer, antioxidant and antimicrobial properties of the sesquiterpene β-caryophyllene from the essential oil of Aquilaria crassna. Molecules.

[35] Marinas I.C., Oprea E., Buleandra M., Badea I.A., Tihauan B.M., Marutescu L., Angheloiu M., Matei E., Chifiriuc M.C. (2021). Chemical Composition, Antipathogenic and Cytotoxic Activity of the Essential Oil Extracted from Amorpha fruticosa Fruits. Molecules.

[36] Chen H., Yuan J., Hao J., Wen Y., Lv Y., Chen L., Yang X. (2019). α-Humulene inhibits hepatocellular carcinoma cell proliferation and induces apoptosis through the inhibition of Akt signaling. Food Chem. Toxicol..

[37] Jang H.-I., Rhee K.-J., Eom Y.-B. (2020). Antibacterial and antibiofilm effects of α-humulene against Bacteroides fragilis. Can. J. Microbiol..

[38] Vukovic N., Milosevic T., Sukdolak S., Solujic S. (2007). Antimicrobial activities of essential oil and methanol extract of Teucrium montanum. Evid. Based Complement. Altern. Med..

[39] Chang K.-F., Huang X.-F., Chang J.T., Huang Y.-C., Lo W.-S., Hsiao C.-Y., Tsai N.-M. (2020). Cedrol, a Sesquiterpene Alcohol, Enhances the Anticancer Efficacy of Temozolomide in Attenuating Drug Resistance via Regulation of the DNA Damage Response and MGMT Expression. J. Nat. Prod..

[40] Liu K., Chen Q., Liu Y., Zhou X., Wang X. (2012). Isolation and biological activities of decanal, linalool, valencene, and octanal from sweet orange oil. J. Food Sci..

[41] Eswaraiah G., Peele K.A., Krupanidhi S., Kumar R.B., Venkateswarulu T. (2020). Identification of bioactive compounds in leaf extract of Avicennia alba by GC-MS analysis and evaluation of its in-vitro anticancer potential against MCF7 and HeLa cell lines. J. King Saud. Univ. Sci..

[42] Jou Y.-J., Hua C.-H., Lin C.-S., Wang C.-Y., Wan L., Lin Y.-J., Huang S.-H., Lin C.-W. (2016). Anticancer activity of γ-bisabolene in human neuroblastoma cells via induction of p53-mediated mitochondrial apoptosis. Molecules.

[43] Li X.-D., Li X., Li X.-M., Yin X.-L., Wang B.-G. (2019). Antimicrobial bisabolane-type sesquiterpenoids from the deep-sea sediment-derived fungus Aspergillus versicolor SD-330. Nat. Prod. Res..

[44] González M.A. (2015). Aromatic abietane diterpenoids: Their biological activity and synthesis. Nat. Prod. Rep..

[45] Pinto M.E., Araujo S.G., Morais M.I., Sá N.P., Lima C.M., Rosa C.A., Siqueira E.P., Johann S., Lima L.A. (2017). Antifungal and antioxidant activity of fatty acid methyl esters from vegetable oils. An. Acad. Bras. Ciências.

[46] Chang W.-T., Liu W., Chiu Y.-H., Chen B.-H., Chuang S.-C., Chen Y.-C., Hsu Y.-T., Lu M.-J., Chiou S.-J., Chou C.-K. (2017). A 4-phenoxyphenol derivative exerts inhibitory effects on human hepatocellular carcinoma cells through regulating autophagy and apoptosis accompanied by downregulating α-tubulin expression. Molecules.

[47] Dimas K., Papadaki M., Tsimplouli C., Hatziantoniou S., Alevizopoulos K., Pantazis P., Demetzos C. (2006). Labd-14-ene-8, 13-diol (sclareol) induces cell cycle arrest and apoptosis in human breast cancer cells and enhances the activity of anticancer drugs. Biomed. Pharmacother..

[48] Mendoza L., Tapia L., Wilkens M., Urzúa A. (2002). Antibacterial activity of 13-epi-sclareol, a labdane type diterpene isolated from Pseudognaphalium heterotrichium and P. cheiranthifolium (Asteraceae). Boletín Soc. Chil. Química.

[49] Selaa F., Karapandzovaa M., Stefkova G., Cvetkovikja I., Trajkovska-Dokikjb E., Kaftandzievab A., Kulevanovaa S. (2015). Antimicrobial activity of berries and leaves essential oils of Macedonian Juniperus foetidissima Willd. (Cupressaceae). Maced. Pharm. Bull.

[50] Liu Y., Zhao L., Ju Y., Li W., Zhang M., Jiao Y., Zhang J., Wang S., Wang Y., Zhao M. (2014). A novel androstenedione derivative induces ROS-mediated autophagy and attenuates drug resistance in osteosarcoma by inhibiting macrophage migration inhibitory factor (MIF). Cell Death Dis..

[51] Xu T., Huang L., Liu Z., Ma D., Zhang G., Ning X., Lu X., Liu H., Jiang B. (2021). Totarol, a natural diterpenoid, induces selective antitumor activity in SGC-7901 human gastric carcinoma cells by triggering apoptosis, cell cycle disruption and suppression of cancer cell migration. J. Buon..

[52] Tavares W.R., Seca A.M. (2018). The current status of the pharmaceutical potential of Juniperus L. metabolites. Medicines.

[53] Yang Y., Yong J. (2018). Chemical and biological progress of Podocarpus nagi. Biomed. Res. Rev..

[54] Okoye T., Akah P., Omeje E., Okoli C., Nworu S., Hamman M. (2011). Antibacterial and anticancer activity of kaurenoic acid from root bark extract of Annona senegalensis. Planta Med..

[55] Abu N., Yeap S.K., Pauzi A.Z., Akhtar M.N., Zamberi N.R., Ismail J., Zareen S., Alitheen N.B. (2016). Dual regulation of cell death and cell survival upon induction of cellular stress by isopimara-7, 15-dien-19-oic Acid in cervical cancer, heLa cells in vitro. Front. Pharmacol..

[56] Morales G., Paredes A., Sierra P., Loyola L.A. (2009). Cytotoxicity, Scavenging and Lipid Peroxidation-Inhibiting Activities of 5, 3´, 4´-trihy-droxy-7-methoxyflavanone Isolated from Haplopappus Rigidus. J. Chil. Chem. Soc..

[57] Ramos A.V., Peixoto J.L., Cabral M.R., Amrein A.M., Tiuman T.S., Cottica S.M., Souza I.M., Ruiz A.L.T., Foglio M.A., Carmo M.R. (2019). Chemical constituents, antiproliferative and antioxidant activities of Vernonanthura nudiflora (Less.) H. Rob. Aerial parts. J. Braz. Chem. Soc..

[58] Stanton R.A., Gernert K.M., Nettles J.H., Aneja R. (2011). Drugs that target dynamic microtubules: A new molecular perspective. Med. Res. Rev..

[59] Mostafa A.E., Atef A., Mohammad A.E.-I., Jacob M., Cutler S.J., Ross S.A. (2014). New secondary metabolites from *Dodonaea viscosa*. Phytochem. Lett..

[60] Kozłowska J., Grela E., Baczyńska D., Grabowiecka A., Anioł M. (2019). Novel O-alkyl derivatives of naringenin and their oximes with antimicrobial and anticancer activity. Molecules.

[61] Imran M., Salehi B., Sharifi-Rad J., Aslam Gondal T., Saeed F., Imran A., Shahbaz M., Tsouh Fokou P.V., Umair M., Arshad H. (2019). Kaempferol: A key emphasis to its anticancer potential. Molecules.

[62] Dawood H.M., Shawky E., Hammoda H.M., Metwally A.M., Ibrahim R.S. (2020). Chemical Constituents from Artemisia annua and Vitex agnus-castus as New Aromatase Inhibitors: In-vitro and In-silico Studies. J. Mex. Chem. Soc..

[63] Yang D., Wang T., Long M., Li P. (2020). Quercetin: Its main pharmacological activity and potential application in clinical medicine. Oxidative Med. Cell. Longev..

[64] Gong G., Guan Y.-Y., Zhang Z.-L., Rahman K., Wang S.-J., Zhou S., Luan X., Zhang H. (2020). Isorhamnetin: A review of pharmacological effects. Biomed. Pharmacother..

[65] Al Bimani B.M.H., Hossain M.A. (2020). A new antimicrobial compound from the leaves of *Dodonaea viscosa*for infectious diseases. Bioact. Mater..

[66] Al-Marzoqi A.H., Hadi M.Y., Hameed I.H. (2016). Determination of metabolites products by Cassia angustifolia and evaluate antimicobial activity. J. Pharmacogn. Phytother..

[67] Haque S., Nawrot D.A., Alakurtti S., Ghemtio L., Yli-Kauhaluoma J., Tammela P. (2014). Screening and characterisation of antimicrobial properties of semisynthetic betulin derivatives. PLoS One.

[68] Yim N.-H., Jung Y.P., Kim A., Kim T., Ma J.Y. (2015). Induction of apoptotic cell death by betulin in multidrug-resistant human renal carcinoma cells. Oncol. Rep..

[69] Ghaneian M.T., Ehrampoush M.H., Jebali A., Hekmatimoghaddam S., Mahmoudi M. (2015). Antimicrobial activity, toxicity and stability of phytol as a novel surface disinfectant. Environ. Health Eng. Manag. J..

[70] Kim D.Y., Choi B.Y. (2019). Costunolide—A bioactive sesquiterpene lactone with diverse therapeutic potential. Int. J. Mol. Sci..

[71] Lin X., Peng Z., Su C. (2015). Potential anti-cancer activities and mechanisms of costunolide and dehydrocostuslactone. Int. J. Mol. Sci..

[72] Guevara Campos B.M.M., Cirio A.T., Galindo V.M.R., Aranda R.S., de Torres N.W., Pérez-López L.A. (2011). Activity against Streptococcus pneumoniae of the essential oil and 5-(3-buten-1-ynyl)-2, 2′-bithienyl isolated from Chrysactinia mexicana roots. Nat. Prod. Commun..

[73] Abraham A., Kattoor A.J., Saldeen T., Mehta J.L. (2019). Vitamin E and its anticancer effects. Crit. Rev. Food Sci. Nutr..

[74] Yenn T.W., Khan M.A., Syuhada N.A., Ring L.C., Ibrahim D., Tan W.-N. (2017). Stigmasterol: An adjuvant for beta lactam antibiotics against beta-lactamase positive clinical isolates. Steroids.

[75] Zhao H., Zhang X., Wang M., Lin Y., Zhou S. (2021). Stigmasterol Simultaneously Induces Apoptosis and Protective Autophagy by Inhibiting Akt/mTOR Pathway in Gastric Cancer Cells. Front. Oncol..

[76] Cao S., Brodie P., Callmander M., Randrianaivo R., Razafitsalama J., Rakotobe E., Rasamison V.E., TenDyke K., Shen Y., Suh E.M. (2009). Antiproliferative triterpenoid saponins of *Dodonaea viscosa* from the Madagascar dry forest. J. Nat. Prod..

[77] Amna T. (2018). Shape-controlled synthesis of three-dimensional zinc oxide nanoflowers for disinfection of food pathogens. Z. Nat. C.

[78] Alqahtani M.S., Al-Yousef H.M., Alqahtani A.S., Rehman M.T., Alajmi M.F., Almarfidi O., Amina M., Alshememry A., Syed R. (2021). Preparation, characterization, and in vitro-in silico biological activities of Jatropha pelargoniifolia extract loaded chitosan nanoparticles. Int. J. Pharm..

[79] Amna T., Alghamdi A.A., Shang K., Hassan M.S. (2021). Nigella Sativa-Coated Hydroxyapatite Scaffolds: Synergetic Cues to Stimulate Myoblasts Differentiation and Offset Infections. Tissue Eng. Regen. Med..

[80] Jorgensen J.H., Hindler J.F., Reller L.B., Weinstein M.P. (2007). New consensus guidelines from the Clinical and Laboratory Standards Institute for antimicrobial susceptibility testing of infrequently isolated or fastidious bacteria. Clin. Infect. Dis..

[81] Alzhrani Z.M.M., Alam M.M., Neamatallah T., Nazreen S. (2020). Design, synthesis and in vitro antiproliferative activity of new thiazolidinedione-1, 3, 4-oxadiazole hybrids as thymidylate synthase inhibitors. J. Enzym. Inhib. Med. Chem..

[82] Ansarali S. (2018). Identification of biological components from potential bone healer medicinal plants. J. Drug Deliv. Ther..

[83] Wollenweber E., Mann K., Yatskievych G. (1986). Epicuticular flavonoid aglycons from leaves of several plants of Mexico and The United States. Bull. Liaison-Groupe Polyphen..

[84] Shalaby N., Abd-Alla H., Hamed M., Al-Ghamdi S., Jambi S. (2012). Flavones composition and therapeutic potential of *Dodonaea viscosa* against liver fibrosis. Int. J. Phytomed..

[85] Amna T., Hassan M.S., Sheikh F.A., Lee H.K., Seo K.-S., Yoon D., Hwang I. (2013). Zinc oxide-doped poly (urethane) spider web nanofibrous scaffold via one-step electrospinning: A novel matrix for tissue engineering. Appl. Microbiol. Biotechnol..

[86] Anandan M., Prabu H.G. (2018). *Dodonaea viscosa* leaf extract assisted synthesis of gold nanoparticles: Characterization and cytotoxicity against A549 NSCLC cancer cells. J. Inorg. Organomet. Polym. Mater..

[87] Alorabi A.Q. (2021). Effective Removal of Malachite Green from Aqueous Solutions Using Magnetic Nanocomposite: Synthesis, Characterization, and Equilibrium Study. Adsorpt. Sci. Technol..

[88] Al-Snafi A.E. (2017). A review on Dodonaea viscosa: A potential medicinal plant. IOSR J. Pharm..

[89] Cao X.T., Showkat A.M., Bach L.G., Lee W.-K., Lim K.T. (2014). Preparation and characterization of Poly (4-vinylpyridine) encapsulated zinc oxide by surface-initiated RAFT polymerization. Mol. Cryst. Liq. Cryst..

[90] Ann L.C., Mahmud S., Bakhori S.K.M., Sirelkhatim A., Mohamad D., Hasan H., Seeni A., Rahman R.A. (2014). Antibacterial responses of zinc oxide structures against Staphylococcus aureus, Pseudomonas aeruginosa and Streptococcus pyogenes. Ceram. Int..

[91] Azam A., Ahmed A.S., Oves M., Khan M.S., Habib S.S., Memic A. (2012). Antimicrobial activity of metal oxide nanoparticles against Gram-positive and Gram-negative bacteria: A comparative study. Int. J. Nanomed..

[92] Stone V., Johnston H., Clift M.J. (2007). Air pollution, ultrafine and nanoparticle toxicology: Cellular and molecular interactions. IEEE Trans. Nanobiosci..

[93] Borm P.J., Robbins D., Haubold S., Kuhlbusch T., Fissan H., Donaldson K., Schins R., Stone V., Kreyling W., Lademann J. (2006). The potential risks of nanomaterials: A review carried out for ECETOC. Part. Fibre Toxicol..

[94] Zhang T., Wang T., Cai P. (2017). Sclareol inhibits cell proliferation and sensitizes cells to the antiproliferative effect of bortezomib via upregulating the tumor suppressor caveolin-1 in cervical cancer cells. Mol. Med. Rep..

[95] de Souza R.A., de Souza Castro M.S., de Souza O.T., Cassio Sola Veneziani R., Kenupp Bastos J., Ambrosio R.S., Alves dos Santos R. (2020). Kaurenoic Acid Induces Cell Cycle Arrest and Apoptosis in the MCF-7 Breast Cancer Cell Line. ChemistrySelect.

[96] Choi J., Lee D.-H., Jang H., Park S.-Y., Seol J.-W. (2020). Naringenin exerts anticancer effects by inducing tumor cell death and inhibiting angiogenesis in malignant melanoma. Int. J. Med. Sci..

[97] Li W., Du Q., Li X., Zheng X., Lv F., Xi X., Huang G., Yang J., Liu S. (2020). Eriodictyol inhibits proliferation, metastasis and induces apoptosis of glioma cells via PI3K/Akt/NF-κB signaling pathway. Front. Pharmacol..

[98] Rajeshkumar S. (2016). Anticancer activity of eco-friendly gold nanoparticles against lung and liver cancer cells. J. Genet. Eng. Biotechnol..

[99] Mishra P.K., Mishra H., Ekielski A., Talegaonkar S., Vaidya B. (2017). Zinc oxide nanoparticles: A promising nanomaterial for biomedical applications. Drug Discov. Today.

