# Supplementary material for: ZnO Nanocomposites of Juniperus procera and Dodonaea viscosa Extracts as Antiproliferative and Antimicrobial Agents
Source: Nanomaterials (Basel). 2022 Feb 16;12(4):664. doi: 10.3390/nano12040664 (PMC8875860; doi:10.3390/nano12040664)
Supplement: Supplementary file 1 [file nanomaterials-12-00664-s001.zip › nanomaterials-1512009-supplementary.pdf]

nanomaterials

Supplementary material for:

ZnO nanocomposites of *Juniperus procera* and *Dodonaea viscosa* extracts as antiproliferative and antimicrobial agents

Maha D. Alghamdi [1], Syed Nazreen [1,*], Nada M. Ali [1] and Touseef Amna [2,*]

[1] Chemistry Department, Faculty of Science, Albaha University, P.O. Box 1988, Albaha 65799, Saudi Arabia; mahaalghamdi@bu.edu.sa (M.D.A.); nada.m@bu.edu.sa (N.M.A.)
[2] Department of Biology, Faculty of Science, Albaha University, P.O. Box 1988, Albaha 65799, Saudi Arabia
* Correspondence: sidrees@bu.edu.sa (S.N.); touseefamna@gmail.com (T.A.)

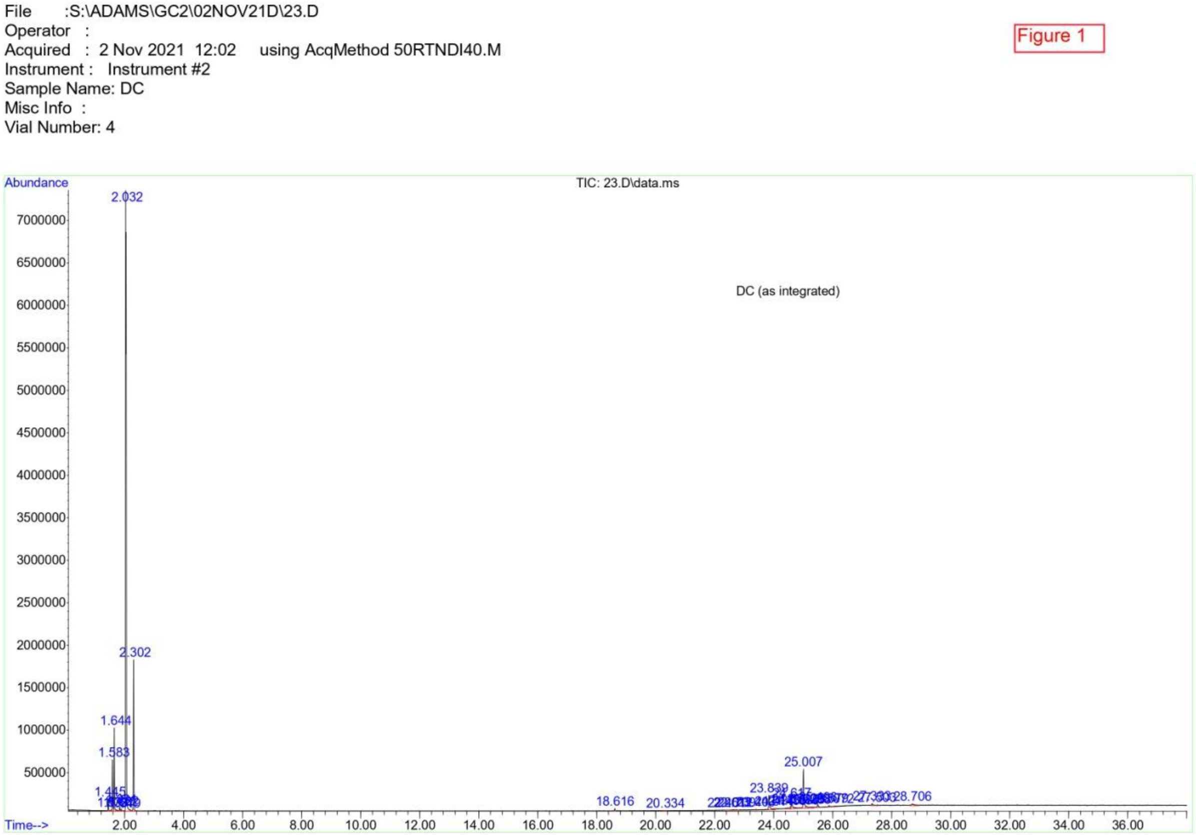

Figure S1. GC-MS chromatogram of *D. viscosa* chloroform fraction.

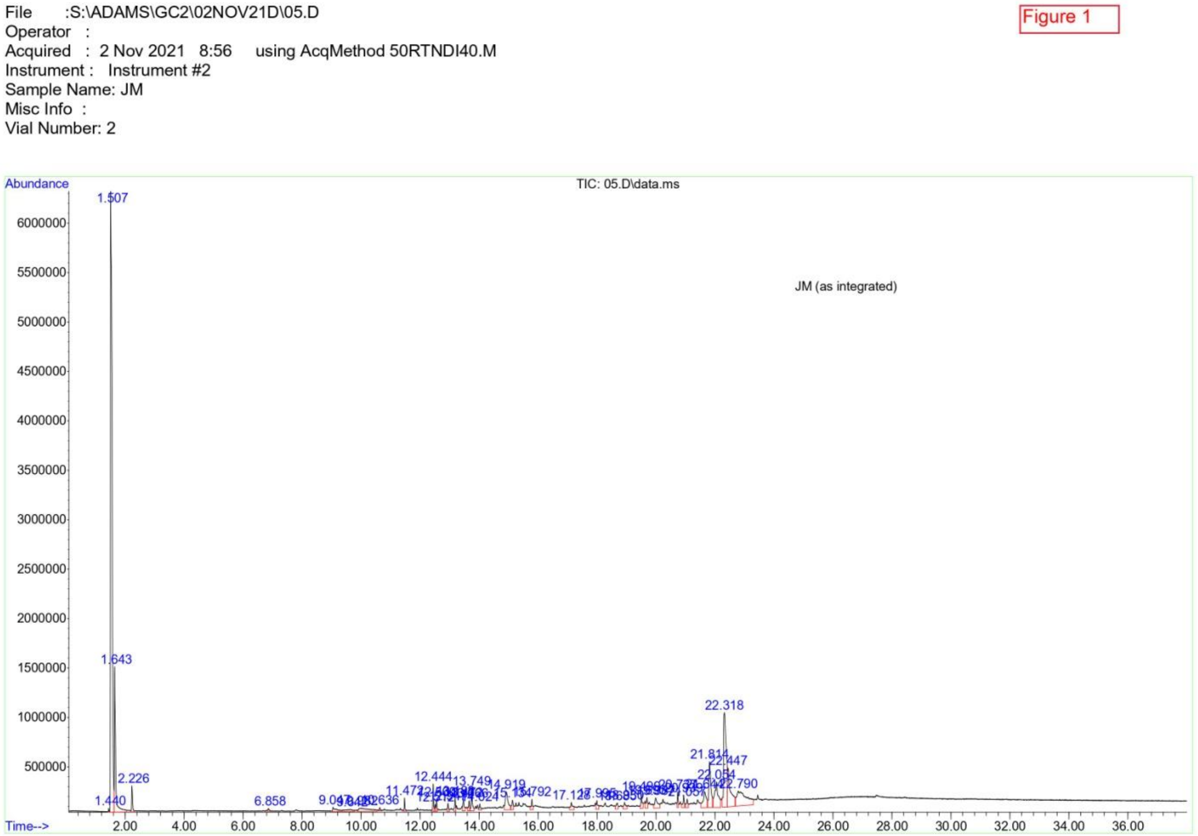

Figure S2. GC-MS chromatogram of *J. procera* methanolic fraction.
